# A high-carbohydrate diet lowers the rate of adipose tissue mitochondrial respiration

**DOI:** 10.1038/s41430-022-01097-3

**Published:** 2022-02-17

**Authors:** Benjamin T. Bikman, Kim J. Shimy, Caroline M. Apovian, S. Yu, Erin R. Saito, Chase M. Walton, Cara B. Ebbeling, David S. Ludwig

**Affiliations:** 1 Department of Cell Biology and Physiology, Brigham Young University, Provo, UT USA; 2 New Balance Foundation Obesity Prevention Center, Boston Children’s Hospital and Department of Pediatrics, Harvard Medical School, Boston, MA USA; 3 Division of Endocrinology, Children’s National Hospital, Washington, DC USA; 4 Division of Endocrinology, Diabetes, and Hypertension, Brigham and Women’s Hospital and Department of Medicine, Harvard Medical School, Boston, MA USA

**Keywords:** Fat metabolism, Obesity

## Abstract

Adipocyte mitochondrial respiration may influence metabolic fuel partitioning into oxidation versus storage, with implications for whole-body energy expenditure. Although insulin has been shown to influence mitochondrial respiration, the effects of dietary macronutrient composition have not been well characterized. The aim of this exploratory study was to test the hypothesis that a high-carbohydrate diet lowers the oxygen flux of adipocyte mitochondria ex vivo. Among participants in a randomized-controlled weight-loss maintenance feeding trial, those consuming a high-carbohydrate diet (60% carbohydrate as a proportion of total energy, *n* = 10) had lower rates of maximal adipose tissue mitochondrial respiration than those consuming a moderate-carbohydrate diet (40%, *n* = 8, *p* = 0.039) or a low-carbohydrate diet (20%, *n* = 9, *p* = 0.005) after 10 to 15 weeks. This preliminary finding may provide a mechanism for postulated calorie-independent effects of dietary composition on energy expenditure and fat deposition, potentially through the actions of insulin on fuel partitioning.

The hormone insulin plays a fundamental role in cellular nutrient signaling, including mitochondrial function. Joslin and Benedict observed in 1912 [1] that the metabolic rate in insulin-deficient states (i.e., type 1 diabetes) is significantly higher than expected—a phenomenon subsequently shown to be correctable with administration of insulin [2]. A component of this change in energy expenditure may involve insulin-induced changes in adipocyte mitochondrial bioenergetics. Dallon et al. [3] found that insulin produces a more tightly coupled state between electron transport and oxidative phosphorylation in rodent brown adipose mitochondria, lowering mitochondrial respiration rate and energy expenditure. Moreover, evidence from rodent models suggests that insulin has a suppressive effect on adipose tissue mitochondrial respiration, thereby lowering adipocyte energy expenditure [4]. While little is known about hormonal and nutrient effects on mitochondrial respiration in human adipose tissue, a recent meta-analysis found that higher- versus lower-carbohydrate diets (which increase insulin secretion) reduce total energy expenditure in humans after 2.5 weeks, with control for energy intake or body weight [5]. The aim of this exploratory study is to test the hypothesis that a high-carbohydrate diet would lower measures of mitochondrial respiration in adipose tissue, consistent with the carbohydrate-insulin model of obesity [6].

## Materials and methods

### Study design

The analyses presented here comprise an ancillary study of the Framingham State Food Study, in which the primary outcome was total energy expenditure [7]. Briefly, adults with BMI ≥ 25 were provided prepared diets at 60% of estimated energy requirements to produce a 10–14% weight loss during the run-in phase. Individuals achieving the target weight loss were weight-stabilized by adjustment of dietary energy and randomly assigned to low- (20%), moderate- (40%), or high- (60%) carbohydrate diets for a 20-week test diet phase. All diets had 20% protein, derived from similar sources across diets, with the remainder of energy derived from fat. Energy provision during the weight-loss maintenance test diet phase was adjusted periodically, targeting ±2 kg of the pre-randomization (post-weight loss) level. Additional detail on diets and experimental design were previously described [7]. A subset of participants in the parent trial was recruited into this ancillary study prior to randomization, protecting against selection bias. Adipose biopsies were obtained following weight stabilization and again after 10–15 weeks on test diets, in conjunction with another ancillary study focused on metabolic fuels [8].

### Participants

Participants in the first two of three cohorts in the parent trial were invited to opt-in, by completing a telephone interview (*n* = 51) followed by an in-person informational session (*n* = 43). After considering additional exclusion criteria (history of allergy or prior reaction to lidocaine, conditions or medications associated with increased risk for bleeding, infection or skin reaction following adipose tissue biopsy, inadequate weight loss in the parent study, and scheduling conflicts with the ancillary study visits), 30 participants opted-in, were eligible for the ancillary study, and were among those randomized to the test diets in the parent trial (see Table 1 for baseline characteristics and weight data). One participant in the low-carbohydrate group did not complete the post-intervention biopsy. Data from two participants were excluded due to loss of pre-intervention biopsy sample (*n* = 1, high-carbohydrate) and development of hypothyroidism (*n* = 1, low-carbohydrate). This report is based on the remaining 27 participants. The study was approved by the Institutional Review Boards at Boston Medical Center and Boston Children’s Hospital, as registered at www.ClinicalTrials.gov (NCT02235038). We obtained written informed consent from participants.

**Table 1:** Participant characteristics (*n* = 27).

Characteristics^a^	Low-carbohydrate group (*N* = 9)	Moderate-carbohydrate group (*N* = 8)	High-carbohydrate group (*N* = 10)
Sex			
Male	5 (55.6%)	4 (50.0%)	3 (30.0%)
Female	4 (44.4%)	4 (50.0%)	7 (70.0%)
Ethnic group			
Hispanic	1 (11.1%)	0 (0.0%)	2 (20.0%)
Non-Hispanic	8 (88. 9%)	8 (100.0%)	8 (80.0%)
BMI, pre-weight loss (kg/m^2^)	31.21 ± 3.69	32.51 ± 4.09	29.97 ± 2.86
Run-in weight loss (kg)^b^	−10.35 ± 2.98	−9.81 ± 2.32	−10.17 ± 2.82
Run-in weight loss (%)^b^	−10.71 ± 1.84	−10.70 ± 1.29	−11.73 ± 1.99
Test phase weight change (kg)^c^	−1.72 ± 2.36	−0.08 ± 2.14	−0.85 ± 1.46
Mitochondrial outcomes (baseline)^d^			
GM	2.17 ± 0.43	2.31 ± 0.40	2.25 ± 0.59
GMD	2.79 ± 0.45	2.90 ± 0.41	2.79 ± 0.46
GMDS	3.23 ± 0.47	3.36 ± 0.50	3.26 ± 0.33
FCCP	4.10 (3.50, 4.10)	4.15 (3.85, 4.25)	4.15 (3.60, 4.30)

^a^For categorical variables, values are frequency (percent). For continuous variables, values are mean ± SD if normally distributed and median (interquartile range) if skewed.

^b^From pre-weight loss to post-weight loss/pre-randomization.

^c^Weight change from post-weight loss/pre-randomization to ~10 weeks of the test diet phase.

^d^First biopsy, post-weight loss/pre-randomization (expressed as pmol O_2_/sec/µg protein). These values provide the baseline for the change results in Fig. 1. GM: glutamate (10 mM) and malate (2 mM); GMD: + ADP (2.5 mM); GMDS: + succinate (2.5 mM); FCCP: + FCCP (0.05 µM).

### Adipose biopsy

Adipose tissue biopsies were performed at Boston Medical Center. Subcutaneous abdominal fat was sampled by needle biopsy before and after the dietary intervention and following an overnight fast. Briefly, a sterile field was prepared, and an area 3 to 5 cm lateral to the umbilicus was isolated, cleaned, and anesthetized with 1% lidocaine. A liposuction cannula (Unitech 1 or 3-hole cannula, 3 mm × 12 cm) was connected to a 60-mL syringe via a 24-inch segment of standard intravenous tubing primed with a small volume of sterile 0.9% normal saline. The cannula was passed through a small cutaneous incision (<0.5 cm) and ~1–3 g of adipose tissue was aspirated under negative vacuum pressure in the 60-mL syringe. Following biopsy, the tissue was frozen in liquid nitrogen and stored at −80 °C. To prepare for mitochondrial analysis, samples were thawed in ice-cold buffer (60 mM K-MES, 35 mM KCl, 7.23 mM K_2_EGTA, 2.77 mM CaK_2_EGTA, 20 mM imidazole, 20 mM tuarine, 5.7 mM ATP, 15 mM PCr, 6.56 mM MgCl_2_−6H_2_O, pH 7.1) and trimmed of connective tissue. Samples were transferred to a tube with chilled buffer and 50 μg/ml saponin, rocked at 4 °C for 30 min, and then washed (105 mM K-MES, 30 mM KCl, 10 mM KH_2_PO_4_, 5 mM MgCl_2_−6H_2_O, 0.5 mg/ml BSA, pH 7.1) at 4 °C for at least 15 min. Samples were stored at −70 °C until assay.

### Mitochondrial respiration assay

Before addition of sample into respiration chambers (Oroboros O2K, Innsbruck, Austria), a baseline respiration rate was determined. After addition of sample, the chambers were hyperoxygenated to ~250 nmol/ml, as previously validated for quantification of mitochondrial respiration rates in human adipose [9]. Respiration was determined by a substrate-uncoupler-inhibitor-titration protocol. Electron flow through complex I was supported by glutamate + malate (10 and 2 mM, respectively) to determine leak oxygen consumption (GM). Following stabilization, ADP (2.5 mM) was added to determine oxidative phosphorylation capacity (GMD). Succinate (2.5 mM) was added to determine complex I + II electron flow into the Q-junction (GMDS). Finally, the chemical uncoupler carbonyl cyanide 4-(trifluoromethoxy) phenylhydrazone (0.05 μM) was added to determine full electron transport system capacity in cells over oxidative phosphorylation (FCCP). Mitochondrial membrane integrity was confirmed in all experiments by observing the effect of adding cytochrome *c* (not shown; 10 μM). Following the protocol, samples were lysed for protein quantification (BCA protein assay; Pierce). Respiration rates are shown relative to total sample protein. Assays were batched by diet group assignments (designated by numerical codes), with laboratory staff masked to codes.

### Statistics

Participant characteristics were summarized with descriptive statistics (frequency and percentage for categorical variables, mean, and standard deviation for continuous variables with normal distribution, median and interquartile range for continuous variables with skewed distribution). Mitochondrial respiration outcomes (pmol O2/sec/ug protein) were calculated as change from first biopsy pre-randomization (post-weight loss) to second biopsy (after 10–15 weeks on weight-loss maintenance test diets). Data were analyzed using general linear models (GLM), with adjustment for sex, ethnicity (Hispanic vs. non-Hispanic) and BMI pre-weight loss. Model assumptions and potential outliers were examined using residual QQ plots and studentized residuals. Statistical analyses were performed using SAS 9.4 and statistical significance was defined as *P* < 0.05 in a two-sided test.

## Results and discussion

**Fig. 1:**
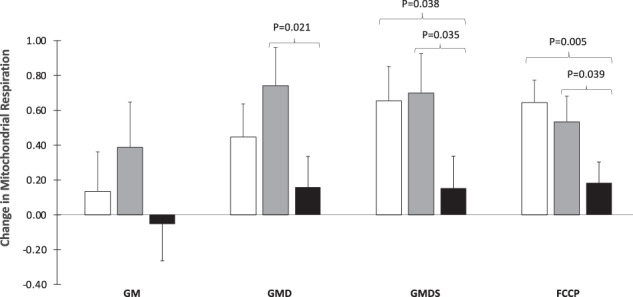
A subcutaneous adipose biopsy was performed in participants 10–15 wk after initiating low- (light bar), moderate- (gray bar), and high- (dark bar) carbohydrate diets with adjustment of energy intake for weight-loss maintenance. Permeabilized adipose samples were sequentially treated with glutamate (10 mM) and malate (2 mM; GM), then ADP (2.5 mM; GMD), then succinate (2.5 mM; GMDS). Finally, FCCP was added (0.05 µM; FCCP). Mitochondrial respiration data (expressed in pmol O2/sec/µg protein) were calculated as change from first biopsy (pre-randomization, post-weight loss) to second biopsy (after 10–15 weeks on weight-loss maintenance diets), as described in Methods.

In the initial condition (GM), an assessment of mitochondrial respiration in a basal state of complex I activation (via glutamate+malate), no differences in mitochondrial respiration were found by diet group. With addition of ADP (GMD) to engage oxidative phosphorylation (via complex V), the high-carbohydrate group had lower respiration compared with the moderate-carbohydrate (*p* = 0.021) but not with the low-carbohydrate group. When complex II-supported respiration was tested with the addition of succinate (GMDS), the high-carbohydrate group had lower respiration versus moderate- (*p* = 0.035) or low- (*p* = 0.038) carbohydrate groups. Finally, the addition of FCCP, which elicits a maximal respiratory response (i.e., mitochondrial uncoupling), produced a robust difference between the high- versus moderate- (*p* = 0.039) or low- (*p* = 0.005) carbohydrate groups (Fig. 1).

Our study suggests that a high-carbohydrate diet, possibly through an increase in insulin secretion [8], lowers mitochondrial respiratory function—a metabolic state that would favor deposition rather than oxidation of fat and predispose to weight gain. This finding is consistent with longer-term feeding trials examining the effects of dietary carbohydrate, as a proportion of total energy intake, on total energy expenditure [5], and a recent study demonstrating adverse effects of carbohydrate overfeeding on cellular redox states [10]. A mechanistic role for insulin secretion on weight gain is supported by a Mendelian randomization study, a two-cohort prospective study, and several clinical trials showing effect modification of dietary carbohydrate by insulin secretion [7, 11–14]. Nevertheless, causal roles for insulin secretion and high glycemic load diets in human obesity have not been established.

As the specific mitochondrial assays were not pre-specified, and the methods have not been standardized among laboratories (including sample storage stability), these findings should be considered preliminary. In addition, we cannot directly translate our findings to whole-body energetics. White adipose tissue is less metabolically active than lean mass, and our sample may not reflect mitochondrial activity in all body fat depots. Nevertheless, even a small shift in substrate partitioning from oxidation to storage, on the order of 1 g body fat per day, could have a major impact on obesity predisposition over the long term. Furthermore, we cannot definitively attribute the observed effects to insulin reduction per se, as other aspects of diet (e.g., fatty acid profile) or indirect influences (e.g., the microbiome) may be mechanistically involved. Additional research is needed to replicate these findings, conduct quantitative energetic studies (e.g., ATP generation), examine generalizability to other populations and experimental conditions, and explore translation to the prevention and treatment of obesity.

## Data Availability

The protocol and dataset are available at Open Science Framework (https://osf.io/rvbuy/).

## References

[1] Benedict FG, Joslin EP. A study of metabolism in severe diabetes. Washington D.C.: Carnegie Institution of Washington; 1912.

[2] Nair KS, Halliday D, Garrow JS (1984). Increased energy expenditure in poorly controlled Type 1 (insulin-dependent) diabetic patients. Diabetologia..

[3] Dallon BW, Parker BA, Hodson AE, Tippetts TS, Harrison ME, Appiah MMA (2018). Insulin selectively reduces mitochondrial uncoupling in brown adipose tissue in mice. Biochem J.

[4] Botezelli JD, Overby P, Lindo L, Wang S, Haida O, Lim GE (2020). Adipose depot-specific upregulation of Ucp1 or mitochondrial oxidative complex proteins are early consequences of genetic insulin reduction in mice. Am J Phys.

[5] Ludwig DS, Dickinson SL, Henschel B, Ebbeling CB, Allison DB (2021). Do lower-carbohydrate diets increase total energy expenditure? An updated and reanalyzed meta-analysis of 29 controlled-feeding studies. J Nutr.

[6] Ludwig DS, Aronne LJ, Astrup A, de Cabo R, Cantley LC, Friedman MI (2021). The carbohydrate-insulin model: a physiological perspective on the obesity pandemic. Am J Clin Nutr..

[7] Ebbeling CB, Feldman HA, Klein GL, Wong JMW, Bielak L, Steltz SK (2018). Effects of a low carbohydrate diet on energy expenditure during weight loss maintenance: randomized trial. BMJ..

[8] Shimy KJ, Feldman HA, Klein GL, Bielak L, Ebbeling CB, Ludwig DS (2020). Effects of dietary carbohydrate content on circulating metabolic fuel availability in the postprandial state. J Endocr Soc.

[9] Walton CM, Jacobsen SM, Dallon BW, Saito ER, Bennett SLH, Davidson LE, et al. Ketones elicit distinct alterations in adipose mitochondrial bioenergetics. Int J Mol Sci. 2020;21. 10.3390/ijms21176255 10.3390/ijms21176255 PMC7503338 32872407

[10] Istfan N, Hasson B, Apovian C, Meshulam T, Yu L, Anderson W (2021). Acute carbohydrate overfeeding: a redox model of insulin action and its impact on metabolic dysfunction in humans. Am J Phys.

[11] Astley CM, Todd JN, Salem RM, Vedantam S, Ebbeling CB, Huang PL (2018). Genetic evidence that carbohydrate-stimulated insulin secretion leads to obesity. Clin Chem.

[12] Wong JMW, Yu S, Ma C, Mehta T, Dickinson SL, Allison DB, et al. Stimulated insulin secretion predicts changes in body composition following weight loss in adults with high BMI. J Nutr. 2021. 10.1093/jn/nxab315. Online ahead of print10.1093/jn/nxab31534587231

[13] Hron BM, Ebbeling CB, Feldman HA, Ludwig DS (2015). Relationship of insulin dynamics to body composition and resting energy expenditure following weight loss. Obesity..

[14] Ebbeling CB, Leidig MM, Feldman HA, Lovesky MM, Ludwig DS (2007). Effects of a low-glycemic load vs low-fat diet in obese young adults: a randomized trial. JAMA..

